# Dynamical feedback between circadian clock and sucrose availability explains adaptive response of starch metabolism to various photoperiods

**DOI:** 10.3389/fpls.2012.00305

**Published:** 2013-01-14

**Authors:** François G. Feugier, Akiko Satake

**Affiliations:** Biosphere Department, Graduate School of Environmental Science, Hokkaido UniversitySapporo, Japan

**Keywords:** carbon starvation minimization, circadian rhythm, phase shift, starch regulation model, carbon partitioning, photoperiod, Arabidopsis, starch and sucrose

## Abstract

Plants deal with resource management during all their life. During the day they feed on photosynthetic carbon, sucrose, while storing a part into starch for night use. Careful control of carbon partitioning, starch degradation, and sucrose export rates is crucial to avoid carbon starvation, insuring optimal growth whatever the photoperiod. Efficient regulation of these key metabolic rates can give an evolutionary advantage to plants. Here we propose a model of adaptive starch metabolism in response to various photoperiods. We assume the three key metabolic rates to be circadian regulated in leaves and that their phases of oscillations are shifted in response to sucrose starvation. We performed gradient descents for various photoperiod conditions to find the corresponding optimal sets of phase shifts that minimize starvation. Results at convergence were all consistent with experimental data: (1) diurnal starch profile showed linear increase during the day and linear decrease at night; (2) shorter photoperiod tended to increase starch synthesis speed while decreasing its degradation speed during the longer night; (3) sudden early dusk showed slower starch degradation during the longer night. Profiles that best explained observations corresponded to circadian regulation of all rates. This theoretical study would establish a framework for future research on feedback between starch metabolism and circadian clock as well as plant productivity.

## Introduction

**Figure 1:**
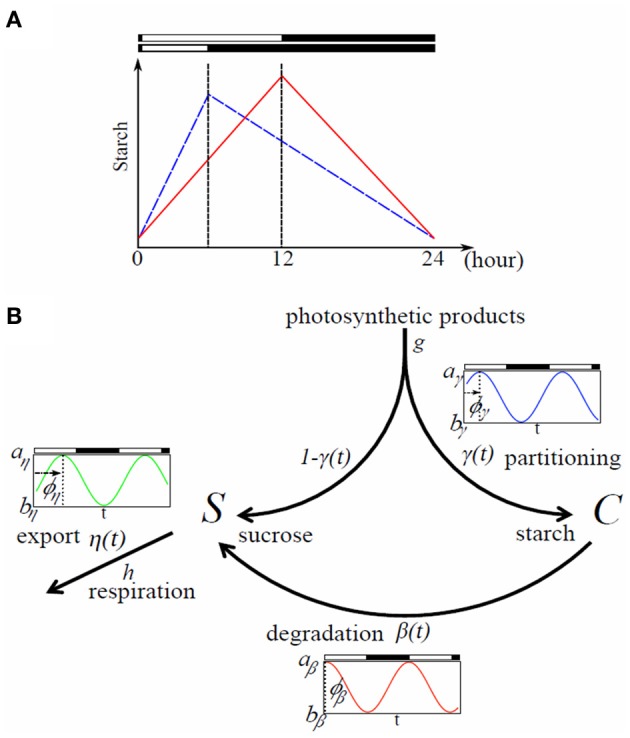
**Typical starch diurnal pattern in a long (LD) and short day (SD) (A) and flow chart of the model (B). (A)** Scheme of starch idealized diurnal pattern in LD (12 h light/12 h dark; red) and SD (6 h light/18 h dark; blue) in plant leaves. The rate of starch synthesis is larger in short days whereas the degradation rate is smaller. This allows the leaves avoiding running out of starch, and therefore sucrose during night. **(B)** Flow chart of the model. Rates γ(*t*), β(*t*), and η(*t*) can be circadian regulated with a phase shift ϕ_*i*_ (*i* ∈ {γ, β, η}); *a*_*i*_ and *b*_*i*_ represent the maximum and minimum values of oscillation of the rate *i*, respectively.

Plants have to deal with resource management to avoid starvation in the dark. During the day they feed on carbohydrates from photosynthesis but need to store substantial amount of photosynthate to sustain metabolism and growth during night. For example, in *Arabidopsis thaliana*, about 50% of the carbon assimilated during the day accumulates as starch in the leaves (Zeeman and Rees, 1999). There is a growing consensus that starch is degraded almost linearly, rather than exponentially (Figure 1A) to provide sugars for growth at night, with 5–10% remaining at dawn (Gibon et al., 2004; Smith and Stitt, 2007; Graf and Smith, 2011). If the night is artificially extended beyond the normal dawn, starch supplies are totally exhausted, which results in carbon starvation indicated by large transcriptional changes (Gibon et al., 2004; Smith and Stitt, 2007). Carbon starvation is one of the factors linked to reduced growth rate, thus it is essential for plants to avoid it (Smith and Stitt, 2007; Yazdanbakhsh et al., 2011; Stitt and Zeeman, 2012).

The length of night varies substantially in seasonal environment. *Arabidopsis* has evolved to avoid carbon starvation by adjusting the rates of starch accumulation and degradation in response to changes in the light/dark (L/D) cycle. Starch degradation speed immediately decreases while its accumulation speed increases (Figure 1A) when the light period is shortened from 16 h light/8 h dark to 8 h light/16 h dark (Lu et al., 2005; Graf et al., 2010) and there are no symptoms of carbon starvation throughout the subsequent longer nights (Graf et al., 2010). This is as if plants anticipate that it is safer to accumulate starch reserves faster during short days and degrade it slower to endure long nights. Further extension of the night to 6 h light/18 h dark cycle led to exhaustion of starch before dawn, but optimal rates of starch metabolism were gradually restored within several days in longer light (Gibon et al., 2004).

Recent reports have demonstrated the importance of circadian clock in regulating carbohydrate assimilation and starch metabolism in order to optimize plant growth in a wide range of L/D cycles (Dodd et al., 2005; Graf et al., 2010; Graf and Smith, 2011; Stitt and Zeeman, 2012). Graf et al. (2010) showed that in wild type *A. thaliana*, starch degradation is programmed to have starch reserves exhausted about 24 h after the last dawn, irrespective of the actual dawn, even in abnormal L/D cycles (such as 14 h light/14 h dark and 10 h light/10 h dark). Further evidence for the involvement of the circadian clock in the control of starch degradation comes from the study of mutants in which the period of the clock is altered. The *cca1 lhy* double mutant is known to have a fast-running circadian clock with a period of about 17 h when placed in constant light. When mutant plants are grown in a 17 h period L/D cycle starch degradation pattern is similar to that of wild-type plants grown in 24 h L/D cycles, while starch is degraded too fast when the mutants are grown under normal 24 h L/D cycles (Graf et al., 2010). When endogenous dawn does not match the actual L/D cycle, growth rate is significantly reduced due to sucrose starvation at the end of the night (Graf et al., 2010) or lower rates of photosynthesis (Dodd et al., 2005). These results indicate that appropriate anticipation of dawn by the circadian clock is essential for optimal growth, resulting in continuous degradation of starch over the course of the night with no symptoms of carbon starvation (Stitt and Zeeman, 2012).

How does circadian clock adjust the rate of starch synthesis/degradation in response to changes in the L/D cycle? Transcripts involved in starch metabolism are rhythmically expressed through L/D cycles (Smith, 2004) and autonomous oscillations persist even in continuous light (Lu et al., 2005) indicating that the clock can affect starch level. In addition, sugar transporters (e.g., putative hexose transporters) are under circadian regulation, peaking late in the subjective light period (Harmer, 2000). These results imply the orchestrated regulation of carbon assimilation, storage, and remobilization by the circadian clock.

*Arabidopsis* circadian system can in turn be regulated by carbohydrates, such as sucrose (Bläsing et al., 2005; James et al., 2008; Dalchau et al., 2011; Haydon et al., 2011). Sucrose is translocated from the shoot to the root and contributes to synchronizing circadian oscillations between these two organs by affecting expression of core oscillator genes *CCA1*, *GI*, and *TOC1* (James et al., 2008). Analysis of global expression profile showed that exogenous sugars cause phase shift of the peaking time of many circadian regulated genes (Bläsing et al., 2005). Exogenous sucrose also alters the period of circadian oscillator both in constant light (Knight et al., 2008) and in constant dark where *GIGANTEA* (*GI*) acts as part of the sucrose-signaling network (Dalchau et al., 2011). These studies suggest that the circadian clock is both regulating and being regulated by starch metabolism in a reciprocal feedback manner. Some modeling studies exploring sucrose and starch patterns (Rasse and Tocquin, 2006) or circadian clock (Edwards et al., 2010) exist, but so far, control of carbon partitioning in plants is not well-known.

Here we hypothesize that the feedback between carbon and circadian clock provides the means to adjust the rates of starch accumulation and consumption in response to changes in the L/D cycle. This hypothesis was tested by a mathematical model assuming that the phase of the circadian clock changes in response to the severity of sucrose starvation. Results of model analysis showed that our feedback hypothesis can explain almost all of the features of starch diurnal profile reported experimentally.

## Methods

### Model for sucrose and starch dynamics in leaves

We build a mechanistic model for sucrose and starch regulation in plant photosynthetic leaves (Figure 1B). The gross production rate of photosyntates is given by *gL*(*t*), where *g* is the constant magnitude rate of photosyntates production and *L*(*t*) is the light availability function taking value 1 for light and 0 for dark. During light period a fraction γ(*t*) of carbon assimilated by photosynthesis is partitioned into starch (*C*)—which accumulates in the leaf through the day—and a fraction 1−γ(*t*) is partitioned into sucrose (*S*)—immediately available for growth. No partitioning occurs at night as there is no photosynthesis. Starch is degraded into sucrose with rate β(*t*) which is the only source of sucrose at night to support leaf respiration and growth. Sucrose is exported with rate η(*t*) to non-photosynthetic tissues such as roots and immature leaves via the phloem. The three rates γ(*t*), β(*t*), and η(*t*) are aggregate parameters of multiple processes (such as cascades of regulations; transcription and translation of various enzymes involved in metabolism) and are treated here as equivalent to the activity of carbon partitioning, activity of starch degradation, and activity of sucrose export, respectively.

Taken together, temporal dynamics of sucrose and starch concentrations in photosynthesizing leaves are represented by the following equations (Figure 1B):

(1) $$\frac{dS}{dt}=(1-\gamma )gL+\beta C-(h+\eta )S,$$

(2) $$\frac{dC}{dt}=\gamma gL-\beta C.$$

Variable *S*, *C*, and the three rates γ, β, and η are functions of the phase of circadian oscillation. Details will be explained later.

The rate of change in sucrose concentration (*S*) in Equation (1) is composed of three terms; the first is equal to the fraction of photosyntate flux that is distributed into sucrose pathway, the second is the flux of sucrose that comes from starch breakdown, and the third is the loss due to leaf maintenance respiration/growth (with constant rate *h*) and export to non-photosynthetic tissues (with rate η(*t*)). Similarly the rate of change in starch concentration (*C*) in Equation (2) is equal to the complementary fraction of the photosyntate flux distributed into starch pathway, minus the loss due to starch breakdown into sucrose for remobilization.

There is increasing evidence that the rate of starch degradation is under circadian control (Dodd et al., 2005; Graf et al., 2010). Thus, we assume β(*t*) to be governed by the internal circadian clock with period τ and formalize it as:

(3) $$\beta_{\phi_{\beta }}\left(t\right)=\left(a_{\beta }-b_{\beta }\right)\left(\cos[2\pi (t-\phi_{\beta })/\tau ]+1\right)/2+b_{\beta }.$$

Parameters *a*_β_ and *b*_β_ represent the maximum and minimum values of oscillations with 0 ≤ *b*_β_ ≤ *a*_β_; ϕ_β_ represents the shift of internal oscillator relative to the external L/D cycle (Figure 1B). When ϕ_β_ = 0, peak of oscillation of β(*t*) is completely synchronized with the initiation of light period. When ϕ_β_ is positive, oscillations of β(*t*) are delayed by an amount ϕ_β_ compared to the external L/D cycle, that is, oscillations of β(*t*) are shifted to the right along the time axis. On the contrary, when ϕ_β_ is negative, oscillations of β(*t*) are advanced by an amount ϕ_β_ and oscillations of β(*t*) are shifted to the left along the time axis.

We also assume that the rate of starch synthesis γ(*t*) and sucrose export η(*t*) are governed by the internal circadian clock with period τ, which is formalized as:

(4) $$\gamma_{\phi_{\gamma }}\left(t\right)=\left(a_{\gamma }-b_{\gamma }\right)\left(\cos[2\pi (t-\phi_{\gamma })/\tau ]+1\right)/2+b_{\gamma },$$

(5) $$\eta_{\phi_{\eta }}\left(t\right)=\left(a_{\eta }-b_{\eta }\right)\left(\cos[2\pi (t-\phi_{\eta })/\tau ]+1\right)/2+b_{\eta }.$$

Similar to Equation (3), parameters *a*_γ_ and *a*_η_ represent the maximum values while *b*_γ_ and *b*_η_ represent minimum values of oscillations for γ(*t*) and η(*t*), respectively. Note that 0 ≤ *b*_γ_ ≤ *a*_γ_ ≤ 1 and 0 ≤ *b*_η_ ≤ *a*_η_ are satisfied; ϕ_γ_ and ϕ_η_ represent the relative shift of each oscillation compared to the external L/D cycle. When *a*_*i*_ = *b*_*i*_, (*i* ∈ {γ, β, η}), there is no circadian oscillation for process *i*.

### Feedback between circadian rhythm and sucrose starvation

**Figure 2:**
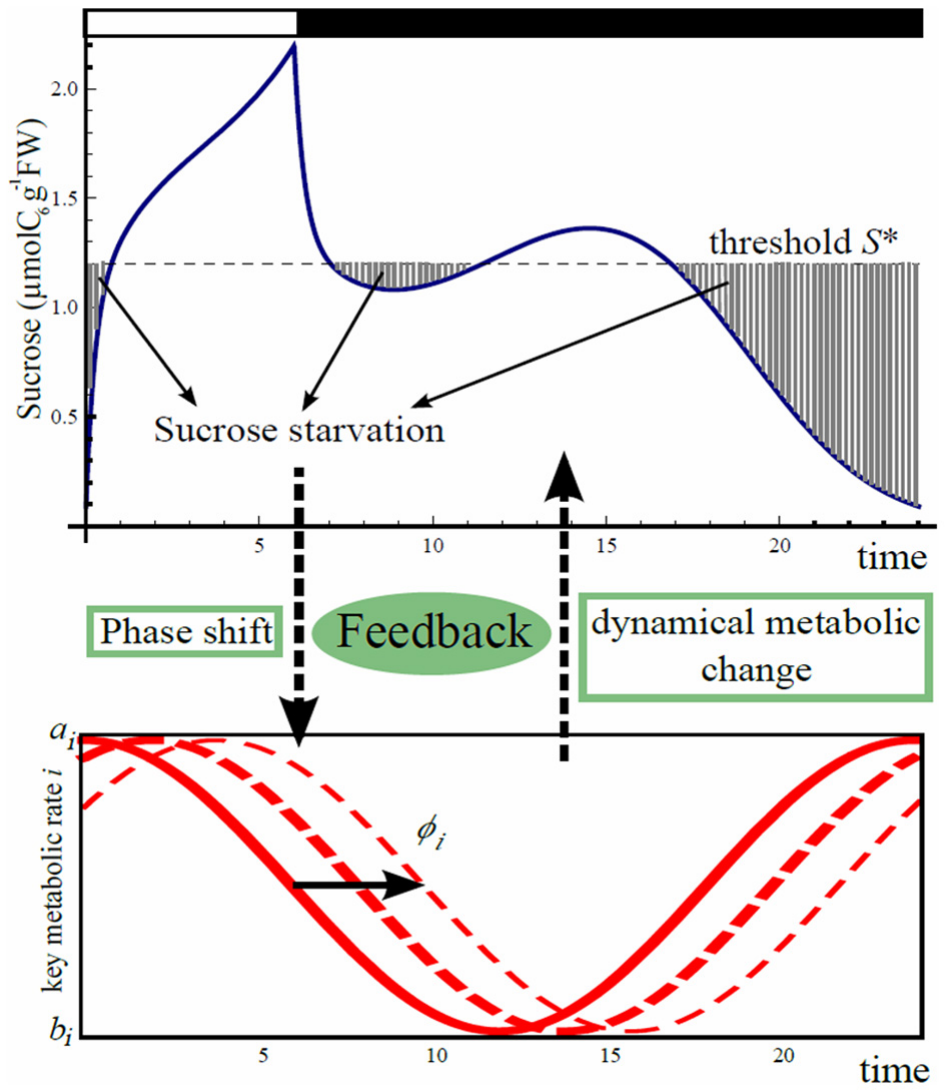
**Scheme of phase shift for starch metabolism and sucrose export driven by carbon starvation.** Carbon starvation occurs when sucrose concentration goes below the threshold S^*^. Starvation is integrated over 24 h from dawn and will induce a shift in time in the oscillation of the three processes, carbon partitioning (γ(*t*)), starch degradation (β(*t*)), and sucrose export (η(*t*)).

Our feedback hypothesis between starch metabolism and circadian clock is formalized by the phase shift in the oscillations of the aforementioned starch metabolic rates and sucrose export rate, in response to the level of sucrose starvation (Figure 2).

We first sought for a set of phase shifts for the three rates that minimizes carbon starvation using gradient descent method. If sucrose drops below the level of requirement S^*^, carbon starvation occurs (Figure 2). We assume that phases of the oscillations of starch metabolic rates and sucrose export rate change independently in response to carbon starvation. These independent feedbacks can be caused by changes of expression of major clock genes, or changes of expression of genes under the clock that directly or indirectly regulate starch metabolism and sucrose transportation. To formalize the phase shift driven by carbon starvation, we first quantitatively define the level of daily average carbon starvation by the following cost function (for a different cost functions refer to Appendix):

(6) $$c\left(\phi_{\gamma },\;\phi_{\beta },\;\phi_{\eta }\right)=\frac{1}{t_{2}-t_{1}}\int_{t_{1}}^{t_{2}}{\left[S^{*}-S_{\phi_{\gamma },\;\phi_{\beta },\;\phi_{\eta }}\left(t\right)\right]}_{+}dt,$$

where S^*^ represents the sucrose concentration threshold below which carbon starvation occurs, and *S*_ϕ_γ_, ϕ_β_, ϕ_η__ (*t*), as in Equation (1), is the sucrose concentration at time *t* for given shift values ϕ_β_, ϕ_γ_, and ϕ_η_. In Equation (6), [*x*]_+_ = *x* when *x* > 0 and 0 otherwise. When *S*_ϕ_γ_, ϕ_β_, ϕ_η__ (*t*) is less than S^*^, the plant is assumed to react to carbon starvation by producing a signal accumulated proportionally to starvation intensity during the time interval *t*_1_ to *t*_2_ assumed to be 24 h counted from dawn.

To find the optimal set of phase shifts that minimizes carbon starvation, we performed a gradient descent of the cost function (6) in phase shift space. Initial shift values were set to 0 for each rate:

(7) $$\phi_{\beta }^{\left(0\right)}=0,\;\phi_{\gamma }^{\left(0\right)}=0,\;\phi_{\eta }^{\left(0\right)}=0,$$

where the suffix in brackets stands for the step number of the gradient descent. Then we solved Equations (1) and (2) numerically for a period of 9 days to remove dependence of sucrose and starch on initial values *S*_ϕ_γ_^(0)^,ϕ_β_^(0)^,ϕ_η_^(0)^_ (0) and *C*_ϕ_γ_^(0)^,ϕ_β_^(0)^_ (0) and obtain a stable rather than transient profile for these two variables. Then we calculated the level of carbon starvation *c*^(*n*)^ [defined in Equation (6)] during the 24 h of the 10th day to obtain the starvation corresponding to the stabilized dynamics. The updated values of the three phase shifts were calculated as follows:

(8) $$\phi_{\gamma }^{\left(n+1\right)}=\phi_{\gamma }^{\left(n\right)}-\varepsilon \frac{\partial }{\partial \phi_{\gamma }}c\left(\phi_{\gamma }^{\left(n\right)},\;\phi_{\beta }^{\left(n\right)},\;\phi_{\eta }^{\left(n\right)}\right),$$

(9) $$\phi_{\beta }^{\left(n+1\right)}=\phi_{\beta }^{\left(n\right)}-\varepsilon \frac{\partial }{\partial \phi_{\beta }}c\left(\phi_{\gamma }^{\left(n\right)},\;\phi_{\beta }^{\left(n\right)},\;\phi_{\eta }^{\left(n\right)}\right),$$

(10) $$\phi_{\eta }^{\left(n+1\right)}=\phi_{\eta }^{\left(n\right)}-\varepsilon \frac{\partial }{\partial \phi_{\eta }}c\left(\phi_{\gamma }^{\left(n\right)},\;\phi_{\beta }^{\left(n\right)},\;\phi_{\eta }^{\left(n\right)}\right),$$

where ε is a small constant. We independently update the value of each phase in the direction decreasing starvation. We repeated the procedure until the gradient descent converges (criterion for convergence is *c*^(*n*)^ − *c*^(*n* + 1)^ ≤ 10^−6^). The set of phase shifts that satisfies this criterion is the optimal set that minimizes the starvation level.

Parameter values of the model, minimum, and maximum for the rate of starch partitioning, starch degradation and sucrose export, gross production rate of photoassimilates and respiration rate were estimated from published data of Gibon et al. (2004) and are shown in Table A1 in Appendix. Data from Gibon et al. (2004) provided good information to estimate all the parameters used in our modeling study. Given that our study focuses on the understanding of the principle governing an observed phenomenon, rather than on precise parameter estimation, one set of data was sufficient. Furthermore, using one set of data guaranties the homogeneity in their acquisition and consistency of units. Model was applied to explain data from experiments where photoperiod was manipulated whereas other parameters such as temperature and nutrient levels were controlled (Gibon et al., 2004). Therefore, we do not consider effects other than those of photoperiod in the model.

## Results

### Starch and sucrose diurnal changes caused by phase shift of circadian clock

**Figure 3:**
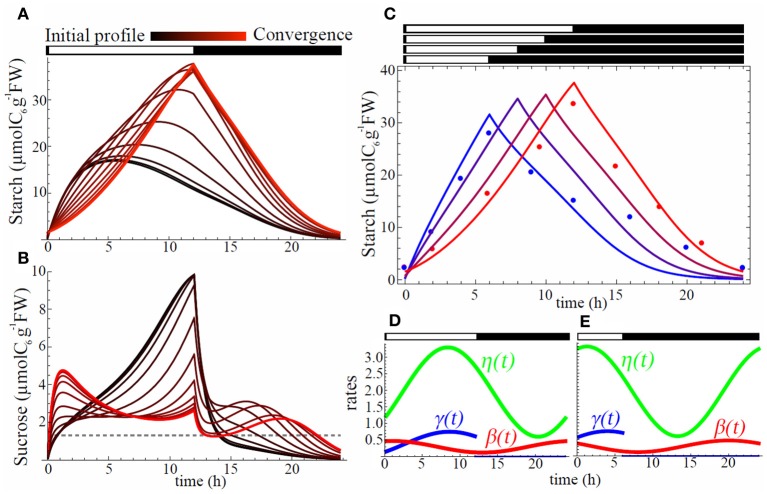
**Starch, sucrose, and rhythm profiles.** Evolution of starch **(A)** and sucrose **(B)** profiles during gradient descent. All of three processes, carbon partitioning (γ(*t*)), starch degradation (β(*t*)), and sucrose export (η(*t*)), are assumed to be circadian regulated. Black color stands for initial profile with all phase shifts equal to 0 whereas red shows profile at convergence of the gradient descent. Starch profiles **(C)** for L/D cycles of 6 h/18 h (blue), 8 h/16 h (purple), 10 h/14 h (dark red), and 12 h/12 h (red). Points are starch data from (2) for SD (blue) and LD (red). Each profile corresponds to an optimal set of phase shifts ϕ_γ_, ϕ_β_, and ϕ_η_ for carbon partitioning (γ(*t*)), starch degradation (β(*t*)), and sucrose export (η(*t*)), respectively, obtained after convergence of the starvation minimization process. Profiles for γ(*t*) (blue), β(*t*) (red), and η(*t*) (green) are shown in **(D)** for LD (12 h/12 h) and in **(E)** for SD (6 h/18 h) conditions.

We first sought for a set of phase shifts that minimizes the level of carbon starvation in 12 h light/12 h dark condition, starting with an initial phase shift value of 0 for each oscillation and initial concentrations for sucrose and starch equal to 0. The initial diurnal pattern of starch was very different from the observed pyramid shape (compare Figures 3A and 1A) and sucrose was severely depleted during night (Figure 3B) revealing that synchrony of the oscillations of the rates with L/D cycle failed to avoid starvation. As gradient descent proceeded, shifting of the phases of the three rates gradually decreased sucrose starvation level and eventually starch diurnal pattern showed almost linear increase during the day and almost linear decrease during night (Figure 3A). This shape closely resembled the one observed in experiments (Gibon et al., 2004) (Figure 3C).

The optimal rate of starch degradation (β(*t*)) was increasing during the night (Figure 3D) to compensate the decreasing starch level. This makes the product β(*t*)C constant, and gives a linear decrease. During the day starch degradation rate was decreasing until dusk in order to replenish the starch pool for the next night. Optimal starch partitioning (γ(*t*)) was low in the morning (Figure 3D) allowing quick replenishment of low sucrose level from the end of the night. It had a peak 3.5 h before dusk allowing a maximum starch accumulation for the upcoming night. Finally optimal sucrose export (η(*t*)) showed the lowest level during the night (Figure 3D) which is effective to avoid starvation.

When the photoperiod was shortened from 12 h light/ 12 h dark to 6 h light/18 h dark, the model successfully reproduced a steeper starch accumulation slope during the day, while slope for degradation was gentler during the night, which is also consistent with observations (Gibon et al., 2004) (Figure 3C). Optimal SD diurnal oscillation patterns (Figure 3E) showed earlier peak (left phase shift) for all three rates. We also ran simulations for other L/D cycles (8 h light/16 h dark and 10 h light/14 h dark) to grasp the progressive change of starch profile over diverse photoperiods. The results showed a smooth transition from the profile obtained in 6 h light/18 h dark condition to the one obtained in 12 h light/12 h dark condition (Figure 3C).

### Necessity of circadian regulation for the avoidance of carbon starvation

**Figure 4:**
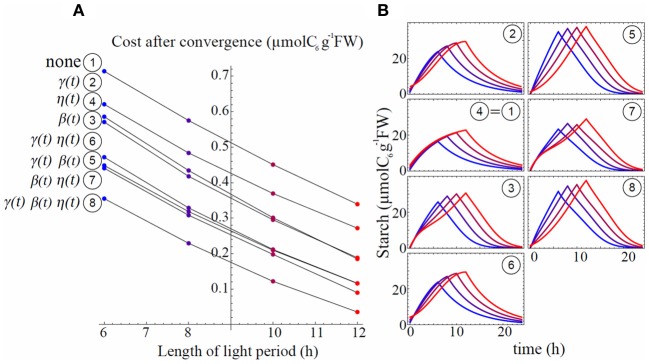
**Starvation cost ranking for all the models (A) with associated starch profiles (B).** L/D cycles are 6 h/18 h (blue), 8 h/16 h (purple), 10 h/14 h (dark red), and 12 h/12 h (red). Starch profiles **(B)** use same color code as in **(A)**. Circled numbers stand for the model number and are ordered following ranking from **(A)** in 6 h/18 h L/D cycle, better models being those with lower costs.

To examine the role of circadian regulation on the three rates (γ(*t*), β(*t*), and η(*t*)) in minimizing carbon starvation, we investigated the situation where either or all of these rates were no longer circadian regulated. We examined the eight different models built up with each combination of constant/oscillating (i.e., no circadian regulation/circadian regulated) rates (Table 1) for each process (see Appendix for parameter estimation for each model). We ranked the models based on their levels of daily mean sucrose starvation after convergence of the gradient descent under four different L/D cycles (Figure 4A). Model 1, in which all rates were assumed constant ($\bar{\gamma }$, $\bar{\beta }$, and $\bar{\eta }$) always gave the worst scores in any L/D cycle condition, implying that either of the three activities needed to oscillate to decrease sucrose starvation. Regardless of the model, the level of daily carbon starvation increased as photoperiod decreased and the ranking was not changed for the different L/D cycles, except for model 2 that performed slightly better than model 3 in 12 h light/12 h dark cycle compared to other L/D cycles. However, in 12 h light/12 h dark cycle light limitation is weak, so is sucrose starvation, hence gradient descent can settle in a wide and shallow “valley.” Therefore, 12 hL/12 hD cycle condition is not severe enough to discriminate which model performs the best. The most effective model to minimize carbon starvation was always model 8 that assumes circadian regulation for all three rates. This implies that circadian regulation of the three processes contributes to decreasing carbon starvation the most efficiently.

**Table 1:** **Combination of oscillating and constant rates used in each of the 8 different models**.

**Model**	**Oscillating**	**Constant**
1	None	$\bar{\gamma }$, $\bar{\beta }$, $\bar{\eta }$
2	γ(*t*)	$\bar{\beta }$, $\bar{\eta }$
3	β(*t*)	$\bar{\gamma }$, $\bar{\eta }$
4	η(*t*)	$\bar{\gamma }$, $\bar{\beta }$
5	γ(*t*), β(*t*)	$\bar{\eta }$
6	γ(*t*), η(*t*)	$\bar{\beta }$
7	β(*t*), η(*t*)	$\bar{\gamma }$
8	γ(*t*), β(*t*), η(*t*)	None

### Triangle shape of starch profile and adaptive response of starch metabolism are explained by circadian control and phase shifting

Starch profiles illustrated in Figure 4B are useful to infer the effect of circadian clock and its phase shifts on starch metabolism (γ(*t*), β(*t*)) and sucrose export (η(*t*)). Starch partitioning (γ(*t*)) has no effect on starch dynamics at night. Similarly, sucrose export (η(*t*)) has no direct effect on the starch dynamics (Equation 2). Thus, starch profile at night only depends on starch degradation rate β(*t*). When starch degradation rate was no longer circadian regulated (models 1, 2, 4, and 6), starch decayed exponentially at night (Figure 4B). However, when β(*t*) oscillated (models 3, 5, 7, and 8) starch showed features of linear decay for a part of the night, similar to the data in Gibon et al. (2004). To obtain such linear starch decay the rate of starch degradation should be accelerating, to counterbalance the decrease in starch concentration. This logic is well-explained by an oscillating β(*t*) with a minimum at dusk.

Models with only one parameter with circadian regulation (models 2, 3, and 4) did not perform well to minimize carbon starvation level, but among the three worst models the one performing the best was generated by oscillating β(*t*) (model 3, Figure 4A). We also notice that among models with two oscillating parameters (models 5, 6, and 7) the best one was characterized by oscillating β(*t*) (model 7). This shows that autonomous rhythm in starch degradation has greater importance than in starch partitioning and sucrose export in order to minimize carbon starvation (models 3, 7, and 8).

The circadian regulation of starch partitioning (γ(*t*)) plays an important role to realize a linear increase in starch level during the day (compare models 1 and 2). However, circadian regulation of γ(*t*) only was not enough to reduce starvation (Figure 4A). The combination of circadian regulation of γ(*t*) and β(*t*) (model 5) could further reduce the level of carbon starvation (Figure 4A), and the resulting starch profile had most of the features observed in the data: linear increase during the day and linear decrease at night. Also, the slope of starch synthesis became steeper as day length decreased, as seen in the observations. However, the slopes for starch decay were all parallel whatever the L/D condition, which is the serious discrepancy between model 5 and observations. Given that γ(*t*) has no effect at night we obtained the same feature of parallel slopes of starch degradation as in model 3.

**Figure 5:**
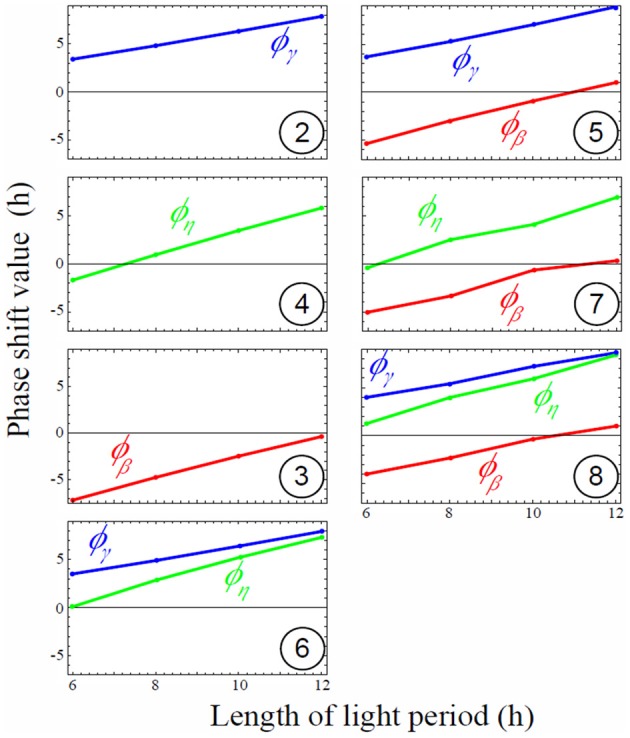
**Magnitude of rate phase shifts for each model depending on light period.** Phase shift of all rates increases linearly (oscillations are delayed compared to the external L/D cycle) as the light period increases. Negative shift values mean oscillations are shifted left (in advance compared to the external L/D cycle), while positive shift values mean oscillations are shifted right (delayed compared to the external L/D cycle). Circled numbers stand for model number. Phase shift values of oscillating rates for the eight models depending on various L/D cycles conditions. Positive values mean right phase shift (or later internal peak) whereas negative values mean left phase shift (or earlier internal peak). No shift (zero) means that the internal circadian peak is synchronized with dawn.

Circadian regulation of sucrose export (η(*t*)) is required to reduce this discrepancy by allowing a decrease in the slope of starch degradation along with a decrease of day length. η(*t*) is involved only in sucrose dynamics. However, it still affects starch profiles since sucrose dynamics modified by η(*t*) affects the value of β(*t*) during convergence of the gradient descent (compare models 3 and 7 in Figure 4B and Figure 5). The effect of η(*t*) on slopes of starch degradation is clearly illustrated in starch profile of model 8, which was the best model minimizing the level of carbon starvation. This model had all of the features of observations, especially different slope values for starch degradation depending on L/D cycles. Indeed, in model 5 the relative phase shift between γ(*t*) and β(*t*) was conserved through the various L/D cycles (Figure 5) giving the identical but shifted starch patterns (Figure 4B). Nonetheless, in model 8 relative phase shifts between η(*t*) and the two other rates were different through the various L/D cycles and slopes of starch degradation became different through the various L/D cycles (Figure 4B).

### Sudden decrease of starch degradation speed in response to unexpected early dusk may not be the result of an instantaneous regulation mechanism

**Figure 6:**
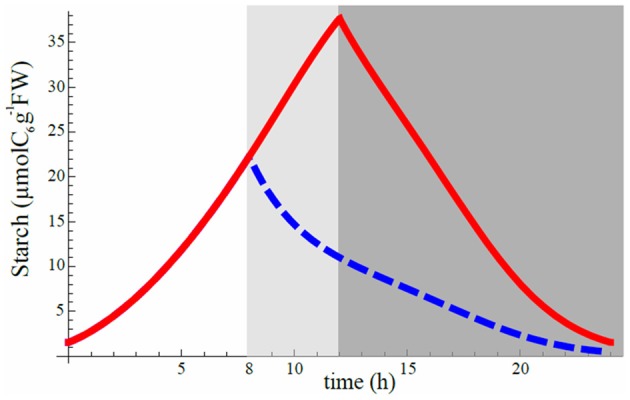
**Starch degradation profile after a sudden shortening of the light period.** Red curve is obtained after minimization of starvation cost and corresponds to the optimal set of phase shifts for 12 hL/12 hD cycle (same as Figure 3C for 12 hL/12 hD cycle). Suddenly shortening the light period to a L/D cycle of 8 h/16 h (extending night period from the dark gray area to the light gray area) while still keeping the optimal phase shift set for 12 hL/12 hD cycle condition gives the blue dashed curve for the first day of the change. Vertical axis scale is perfectly consistent with Figure 1A of Graf et al. (2010) when converting their scale by taking 1 mg of *C*_6_ to be equal to 5.85 μmol.

It was shown that plants subject to an unexpected early dusk surprisingly exhibited a lower starch degradation speed than those with normal dusk as if the plant reacted instantly to a shorter day, prelude to a probable longer night, by saving starch (Lu et al., 2005; Graf et al., 2010; Graf and Smith, 2011). Our model could reproduce the same feature as the observations (Figure 6). We chose the best set of phase shifts in 12 h light/12 h dark cycle for model 8 and run a simulation in this L/D cycle. Then we suddenly changed the L/D cycle to 8 h light/16 h dark to predict how starch profile would be affected. The model was able to reproduce a sudden decrease of starch degradation speed during the longer night (Figure 6). The predicted decline of starch degradation is independent of any dynamical adjustments of starch degradation activity at the moment of (or consequent to) the early dusk, for a longer upcoming night. It is, in fact, explained by the lower starch concentration at early than at normal dusk. Given that the degradation speed of starch is the product of the lower starch level at early dusk by the relatively unchanged degradation rate β(*t*) (Figure 3D) in Equation (2) (βC), the product is smaller during the whole night, so the reaction speed is slower, hence starch degradation has a milder slope. As shown in Figure 4B (models 1 2, 4, and 6), a constant rate of starch degradation cannot reproduce linear decay of starch during night. Thus, a slower and linear decrease in starch level after a sudden early dusk may not be the result of a sophisticated regulation mechanism. For quantitative explanation of experimental data (Lu et al., 2005; Graf et al., 2010), further experimental and theoretical studies are needed.

## Discussion

The presented model, with simple assumptions of circadian regulated rates and starvation avoidance by shifting of oscillation phase, could reproduce most of the features observed in experiments. While most biological quantities follow exponential behavior, the linear decrease (increase) of starch concentration at night (in the light) in *Arabidopsis* leaves is rather unusual. Nevertheless, by using a sinusoidal degradation (partitioning) rate for starch we could obtain such linear decrease (increase). Furthermore, shifting of the internal phase of oscillations of the key metabolic activities was enough to reproduce observations, namely a faster starch accumulation in shorter light period followed by a slower degradation during longer dark period, while keeping linear features in both cases. As a result of this behavior sucrose starvation was decreased.

The model could also explain the reduced degradation speed of starch in case of sudden early dusk. In fact, this behavior may not be the result of a sophisticated regulation from the plant but simply the result of a lower starch level at the time of early dusk for a similar value of the degradation rate. Although this behavior is not the result of any regulation from the plant it offers the advantage of saving starch.

As for the circadian oscillator, our choice of the cosine function to represent circadian oscillations may introduce a bias in our results, particularly in the profile of starch, because it is a simplification of actual biological oscillators. To obtain a linear decay of starch at night the degradation rate β(*t*) should follow an accelerating increase. Starch decay will diverge from linear otherwise (Figure 3C). Including the dynamical system of the circadian clock with the effect of sucrose on the clock period in our model would help to draw more precise predictions, and would also be helpful to explore the mechanism of phase shift observed in empirical works (Bläsing et al., 2005) and which remains as a black box in this study. Indeed, although simulation results agree with data we still need to understand how the plant knows the magnitude and direction of the shifts.

There are many possibilities to extending our model. Regarding the aggregate parameter β(*t*) we used a minimum number of assumptions making it time (clock)-dependent only. However, it can include more detailed dynamics such as saturation by high starch level. If the surface of a starch granule can host all the enzymes degrading it into sucrose, the degradation would occur at a constant rate until the moment the granule surface is too small to host all the enzymes. In this case β(*t*) would be proportional to the enzyme level and inverse proportional to *C*.

In our results we noticed that circadian regulation of all rates gave the lowest sucrose starvation. However, it is possible that other parameters of the model change with time or with other variables independently from the clock. For example, export rate of sucrose from the leaf may be inhibited by low sucrose level to prevent starvation in the exporting organ. However, with such regulation the profile of sucrose export should be close to the profile of η(*t*) obtained in our simulations.

In the optimization procedure we considered an objective function including only cost of sucrose starvation. It is likely that plants may try to maximize growth at the same time by supplying growing organs with a constant flux of sucrose. To do so, one can modify the objective function in the present model by including other terms such as sucrose export. Thus, plants would maximize sucrose export to growing organs while avoiding depleting sucrose level in exporting organs.

However, it is interesting to notice that minimization of sucrose starvation, an intuitively critical quantity for plant growth and survival, could recreate seemingly complicated behaviors observed in starch profiles under various photoperiods. This choice of modeling offers valuable information regarding how the metabolic network of plants may have been shaped by natural selection to optimize resource management. That is the type of metabolisms that remain nowadays.

Nevertheless, because of its simplicity, the present model helps understanding the mechanism of adaptive starch management behind the seeming complicated starch and sucrose behaviors. Proper management of starch allows suitable export of sucrose to other organs, which significantly influences the productivity of plants. Thus, a better understanding of starch management would have important consequences for crop performance and plant evolution. In this direction, further interaction between empirical and theoretical studies is essential.

## Author contributions

Akiko Satake proposed the model. François G. Feugier and Akiko Satake discussed and improved the model together. François G. Feugier wrote the programs, fitted the data, and ran the simulations to produce the results. Both authors discussed the results and were involved in the manuscript preparation.

### Conflict of interest statement

The authors declare that the research was conducted in the absence of any commercial or financial relationships that could be construed as a potential conflict of interest.

## Figures and Tables

**Figure A1:**
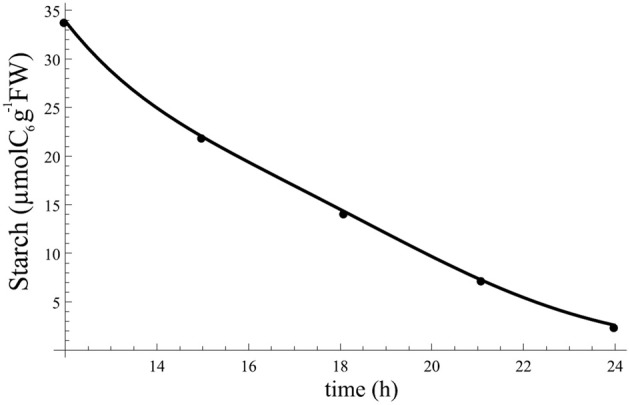
**Starch profile at night resulting from fitting Equation (A1) to data and using β(*t*).** Points indicate the data to which Equation (A1) was fitted.

**Figure A2:**
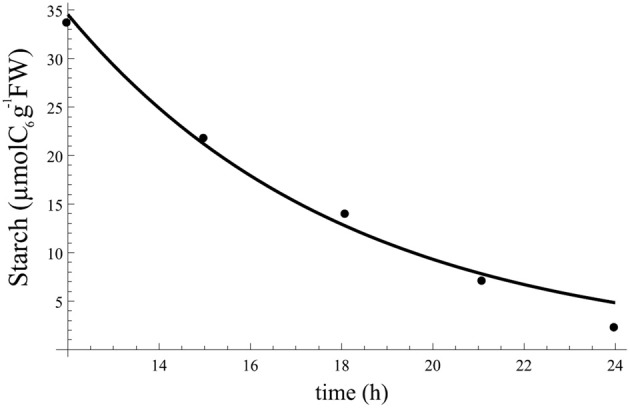
**Starch profile at night resulting from fitting Equation (A1) to data and using *a*_β_ = *b*_β_ = 0.16.** Points indicate the data to which Equation (A1) was fitted.

**Figure A3:**
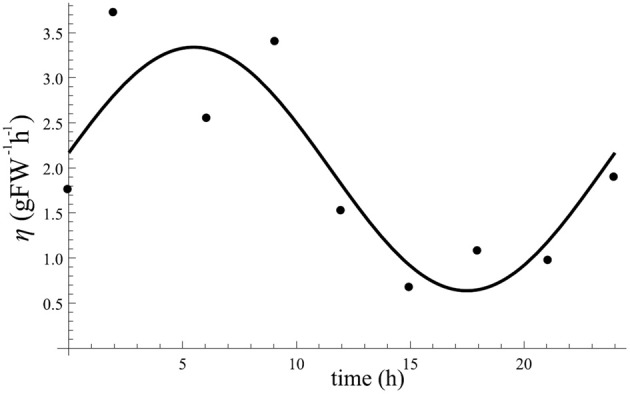
**Profile of oscillating η(*t*) after fitting to estimates (points) of η(*t*).** Obtained fitting values are *a*_η_ = 3.3/h, *b*_η_ = 0.6/h, and ϕ_η_ = 5.5 h.

**Figure A4:**
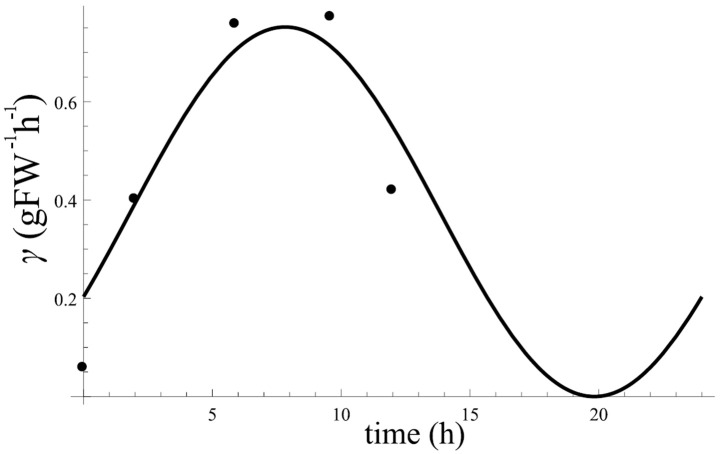
**Estimated point values for γ at each sample data from Figure 2A from Gibon et al. (2004) using oscillating β(*t*).** The curve is the result of fitting a cosine to the estimation points of γ. From the fitting we obtain *a*_γ_ = 0.75, *b*_γ_ = 0, and ϕ_γ_ = 7.8.

**Figure A5:**
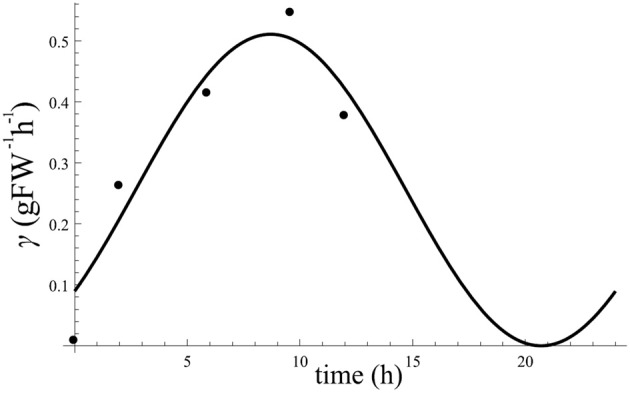
**Estimated point values for γ(*t*) at each sample data from Figure 2A using constant $\bar{\beta }$.** The curve is the result of fitting a cosine to the estimation points of γ(*t*). By fitting a cosine function to the obtained estimations for γ(*t*) we obtain *a*_γ_ = 0.51, *b*_γ_ = 0, and ϕ_γ_ = 7.8.

**Figure A6:**
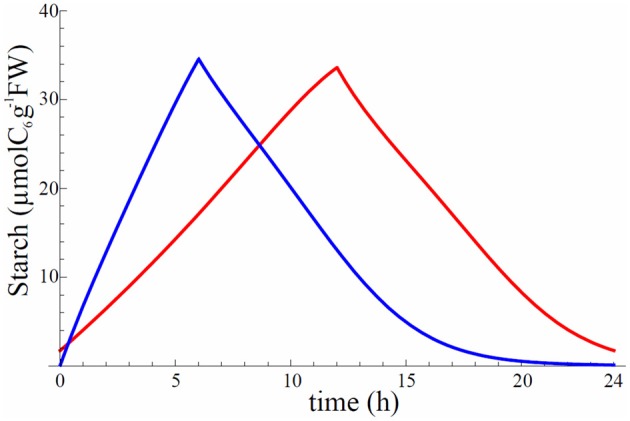
**Starch profile for model 8 after convergence of gradient descent using homeostasis cost function**. Red is for 12 h/12 h and blue is for 6 h/18 h L/D cycle.

## Appendix

### Parameters estimation for method section

The model contains 16 parameters as summarized in Table A1. We estimated all of these parameter values from the data published in Gibon et al. (2004). Thus, when we mention about data sources (such as Figures 2 or 3), all of them come from the study by Gibon et al. (2004). The rates for starch partitioning (γ), starch degradation (β), and sucrose transportation (η) were assumed either circadian regulated with period τ, or not circadian regulated and are given as constants. Data from Gibon et al. (2004) were extracted by measuring the printed data by hand with a ruler. The method of fitting used in the following is “FindFit” from Mathematica 8.0. The period of oscillation (τ) is fixed as 24 h.

**Table A1:** **Parameter estimates**.

**Symbol**		**Description**		**Units**	**Value**	
*g*		Carbon fixation by photosynthesis		μmolC_6_/gFW/h	12.7	
*h*		Respiration		/gFW/h	0.79	
*s*^*^		Starvation threshold		μmolC_6_/gFW	1.3	
τ		Light/dark cycle period		hour	24	
η		Sucrose export rate			η(*t*) Oscillating	$\bar{\eta }$ Constant
*a*_η_		Maximum export rate		/gFW/h	3.3	1.98
*b*_η_		Minimum export rate		/gFW/h	0.6	1.98
γ(*t*)		Carbon partitioning assumed oscillating		Dimensionless		
*a*_γ_		Maximum partitioning fraction when oscillating		Dimensionless		
*b*_γ_		Minimum partitioning fraction when oscillating		Dimensionless		
$\bar{\gamma }$		Carbon partitioning assumed constant		Dimensionless		
β(*t*)		Starch degradation rate assumed oscillating		/gFW/h		
*a*_β_		Maximum Starch degradation rate when oscillating		/gFW/h		
*b*_β_		Maximum Starch degradation rate when oscillating		/gFW/h		
$\bar{\beta }$		Starch degradation rate assumed constant		/gFW/h		
		**γ(*t*)**	**$\bar{\gamma }$**			
β(*t*)	*a*_β_	0.47	0.47			
	*b*_β_	0.12	0.12			
	*a*_γ_	0.75	0.49			
	*b*_γ_	0	0.49			

#### Estimation of the starch degradation rate, β(*t*)

***Circadian regulated rate β(*t*)***. When starch degradation rate oscillates with period τ, β(*t*) is given as:

(A1) $$\beta (t)=(a_{\beta }-b_{\beta })(\cos[2\pi (t-\phi_{\beta }(t))/\tau +1])/2+b_{\beta }.$$

We estimated *a*_β_, *b*_β_, and ϕ_β_, maximum, minimum, and the phase shift of the oscillations, respectively, by fitting Equation (A1) to the night data representing starch profile during long day condition (Figure 2A in Gibon et al., 2004). At night the dynamics of starch (*C*) are independent of the dynamics of sucrose (*S*) and inflow from photosynthesis. Therefore, the dynamics of *C* at night are given as:

(A2) $$\frac{dC}{dt}=-\beta (t)C$$

which is simple enough to be fitted to data. The parameter values obtained from the fitting are *a*_β_ = 0.47 μmolC_6_/gFW/h, *b*_β_ = 0.12 μmolC_6_/gFW/h and ϕ_β_ = 3.2 h, and the resulting function for β(*t*) is illustrated in Figure A1.

***Constant*$\bar{\beta }$**. We fitted the data using Equation (A1) under constraint of *a*_β_ = *b*_β_, and obtained *a*_β_ = *b*_β_ = 0.16. When using this constant value of $\bar{\beta }$ in the dynamics of starch at night [Equation (A1)] we obtain the profile in Figure A2 for starch at night.

#### Estimation of net carbon fixation rate g and respiration rate h

We estimated the values of *g* and *h* from Figures 2A and 3A representing the rate of carbon fixation during 24 h. At night, the negative values of carbon fixation give the intensity of respiration alone, that is:

$$\text{carbon fixation during night}=-hS_{6}$$

where *S*_6_ is the concentration of sucrose measured in six carbon units. The carbon fixation during night was estimated from Figure 3A in Gibon et al. (2004) as −1.5 μmolC_6_/gFW/h. In contrast, during the day the net carbon fixation is equal to photosynthetic rate minus the carbon lost by respiration. That is:

$$\text{carbon fixation during day}=g-hS_{6}.$$

Therefore, the net carbon fixation rate is given as:

(A3) $$g=\text{carbon fixation during day}+hS_{6}.$$

The carbon fixation during day was estimated from Figure 3A as 11.2 μmolC_6_/gFW/h. Using Equation (A3), we estimated the net carbon fixation rate as *g* = 11.2 + 1.5 = 12.7 μmolC_6_/gFW/h.

The value of *S* at night needed to estimate *h*, was estimated as *S* = 1.9 μmolC_6_/gFW by averaging the values of sucrose concentration at night from Figure 2B. Finally we obtain *h* = −1.5/(−1.9) = 0.79/h.

#### Estimation of sucrose exportation rate η

***Circadian regulated rate η(*t*)***. In our model sucrose export appears in Equation (1) as the term η(*t*)*S*. To find the value of η(*t*) we need to know *S* during 24 h. Information for *S* is available from Figure 2B, while the data in Figure 3B provide information of the export rate of sugar during 24 h. We therefore use the data from both figures at joint sampling times to calculate the value of η(*t*) at each of these sampling times. The resulting estimation points for η(*t*) are plotted in Figure A3.

We then fitted a cosine function for maximum value of *a*_η_, minimum value of *b*_η_, and phase shift of ϕ_η_. The result of data fitting is also shown in Figure A3. Fitted values obtained are *a*_η_ = 3.3/h, *b*_η_ = 0.6/h, and ϕ_η_ = 5.5 h.

***Constant*$\bar{\eta }$**. To obtain the value of $\bar{\eta }$ as a constant we calculated the mean value of the estimated η(*t*) that is circadian regulated (Figure A3) and found $\bar{\eta }=2.0/h$.

#### Estimation of carbon partitioning rate γ

To estimate γ we used the simplest equation of the model containing it, which is Equation (1) in the main text:

(A4) $$\frac{dC}{dt}=g\gamma \left(t\right)-\beta \left(t\right)C$$

The problem is that Equation (1) also contains β that we estimated earlier. We previously assumed that β can be either circadian regulated or constant, and we also used these two assumptions for γ. Therefore, we needed to calculate the four different possibilities for γ: circadian regulated γ(*t*) with β circadian regulated or constant; constant $\bar{\gamma }$ with β circadian regulated or constant.

***Circadian regulated* γ(*t*) and β(*t*)**. The positive slope of the curve in Figure 2A represents the rate change of starch (*d*C/dt) during day. As formalized in Equation (1) in the main text, *d*C/dt is given by the input term from photosynthesis *g*γ where *g* was estimated earlier; and the degradation term of starch βC where β has also been estimated earlier as circadian regulated or constant; the level of starch *C* is shown on Figure 2A. Hence the only unknown parameter is γ. Therefore, we solved Equation (1) in the main text to estimate γ using each sample data available from Figure 2A. The resulting points for γ estimations are shown in Figure A4. We fitted a cosine function to the data points estimated for γ(*t*). From the fitting we obtain *a*_γ_ = 0.75, *b*_γ_ = 0, and ϕ_γ_ = 7.8.

***Constant*$\bar{\gamma }$ with β(*t*) *circadian regulated***. We calculated the mean value of the estimated γ(*t*) that is circadian regulated (Figure A4) and found $\bar{\gamma }=0.49$.

***Circadian regulated* γ(*t*) *with*$\bar{\beta }$*constant***. Here we solve Equation (1) from the main text with constant $\bar{\beta }$ (found in section “Constant $\bar{\beta }$”) to estimate γ(*t*) at each sample point of *C* obtained from Figure 2A, and results were plotted in Figure A5. We then fitted a cosine function with maximum value of *a*_γ_, minimum value of *b*_γ_ and phase shift of ϕ_γ_ to these points. The result of data fitting is shown in Figure A5. We obtained *a*_γ_ = 0.51, *b*_γ_ = 0 and ϕ_γ_ = 7.8.

***Constant*$\bar{\gamma }$*with*$\bar{\beta }$*constant***. To obtain the value of $\bar{\gamma }$ as a constant under constant $\bar{\beta }$ we calculated the mean value of the estimated γ(*t*) that is circadian regulated (Figure A5) and found $\bar{\gamma }=0.33$.

#### Estimation of the sucrose starvation level s^*^

Finally, in the present model the main assumption is that there is a sucrose concentration *s*^*^ below which plants start to starve. By looking at data from Figure 2B we see that the lowest sucrose concentration occurs at the end of the night and is equal to 1.3 μmolC_6_/gFW. Given that the experiment is in long day condition the plant may not experience any starvation at night and 1.3 μmolC_6_/gFW may be a safe concentration for the plant. However, below this threshold plant may start to experience starvation. Hence we decided to set the level of starvation *s*^*^ at 1.3 μmolC_6_/gFW.

### Other starvation cost function: homeostasis cost function

To check the robustness of the results regarding the cost function we tried the following homeostasis cost function:

$$c\left(\phi_{\gamma },\;\phi_{\beta },\;\phi_{\eta },\right)=\frac{1}{t_{2}-t_{1}}\int_{t_{1}}^{t_{2}}{\left(S^{*}-S\left(t,\;\phi_{\gamma },\;\phi_{\beta },\;\phi_{\eta }\right)\right)}^{2}dt.$$

This cost function brings a penalty to the cell when sucrose concentration is not exactly equal to *S*^*^, whereas starvation cost function penalizes the cell only when sucrose concentration is below *S*^*^. This homeostasis cost function tends to create a flat sucrose profile the closest possible to *S*^*^. Simulation with model 8 using homeostasis cost function in 12 hL/12 hD cycle gave similar results to those when using the starvation cost function (Figure A6). However, in 6 hL/18 hD cycle condition results were closer to those from model 5, with a fast starch increase during the day and a degradation speed equal to the one in 12 hL/12 hD cycle condition (parallel degradation curves for both L/D cycle conditions). Also, the peak of starch at dusk in 6 h/12 h is larger than the one in 12 hL/12 hD cycle, which is inconsistent with the data and does not improve prevention of starvation. For these reasons we decided to discard the homeostasis cost function which is more restrictive in the condition of the present model, and kept the starvation cost function which sounds more natural as a first principle.
